# What makes children learn how to swim? – health, lifestyle and environmental factors associated with swimming ability among children in the city of Malmö, Sweden

**DOI:** 10.1186/s12887-021-03094-0

**Published:** 2022-01-10

**Authors:** Mare Lõhmus, Mehdi Osooli, Frida I. H. Pilgaard, Per-Olof Östergren, Anna Olin, Stefan Kling, Maria Albin, Jonas Björk

**Affiliations:** 1 Division of Occupational and Environmental Medicine, Lund University, Lund, Sweden; 2 Institute of Environmental Medicine, Karolinska Institute, Stockholm, Sweden; 3 Centre for Occupational and Environmental Medicine, Stockholm, Region Stockholm Sweden; 4 Center for Primary Health Care Research, Department of Clinical Sciences, Lund University, Malmö, Sweden; 5 Division of Social Medicine and Global Health, Department of Clinical Sciences, Lund University, Malmö, Sweden; 6 Primary School Administration, Department of Student Health, Malmö, Sweden; 7 Department of Child and Adolescent Psychiatry, Lund University Hospital, Lund, Sweden; 8 Unit of Occupational Medicine, Institute of Environmental Medicine, Karolinska Institute, Stockholm, Sweden; 9 Clinical Studies Sweden, Forum South, Skåne University Hospital, Lund, Sweden

**Keywords:** Socioeconomic factors, Exercise, Swimming, Wellbeing, Social support

## Abstract

**Background:**

Swimming ability among children in the city of Malmö, Sweden is strongly affected by socioeconomic differences. We investigated to what extent mediating health and lifestyle factors, such as children’s eating, sleeping and physical activity habits, as well as the characteristics of the social and working environment at both school and home, could explain the socioeconomic gradient in swimming ability.

**Methods:**

Our study population included children who started their first-grade school-year in 2012 or 2013 at any of the public primary schools of Malmö, Sweden. Cross-sectional, self-reported questionnaire-based data about health status and swimming ability in the fourth grade (age 10) were included from the Pupil Health Database (ELSA) for 3468 children.

**Results:**

Children’s self-reported swimming ability was strongly associated with both individual- and school-based sociodemographic variables. Nine health, lifestyle and environmental variables were identified as potential mediators and included in the final model. Four of these variables, “Activity”, “Outdoor time”, “Social relationships at home and on the free time”, and “Positivity about future”, were significantly and positively associated with children’s ability to swim.

**Conclusions:**

Social support, optimism for the future and an active lifestyle were positively associated with children’s swimming skills; however, compared to the socioeconomic factors, these health- and lifestyle factors contributed very little. It is possible, that interventions concerning children’s swimming ability in lower socioeconomic neighbourhoods, should in addition to children’s swimming lessons, target the whole families with the goal of increasing their possibilities for socialising and engaging in different kinds of recreational activities.

**Supplementary Information:**

The online version contains supplementary material available at 10.1186/s12887-021-03094-0.

## Background

Swimming is a potentially life-saving skill and learning to swim in an early age may reduce the numbers of children drowning [1, 2]. Swimming education in Sweden is part of the primary school curriculum and passing a swimming test is one of the requirements for being approved in the health and physical education course of the school years six and nine [3]. To pass the obligatory swimming tests at primary school, children in Sweden have to be able to swim continuously for 200 m, of which at minimum 50 m on backstroke [4]. Failing the swimming test in ninth grade may results in incomplete school grades and have a negative impact on the individual’s chances to enter upper secondary school programmes.

Despite it being part of the school curriculum, large differences in swimming ability in children exist between different Swedish municipalities, with low swimming ability being more common in areas with a high proportion of families with a low socioeconomic status and immigrant background [4–6]. According to a case analyse of all child-drownings in Sweden, between 1998 and 2007, children from single parent families and with immigrant background had a higher risk of drowning than children with Swedish origin and from two parents families [7]. In a recent article, Pilgaard et al. 2019 [4] investigated whether the introduction of a community-level swimming intervention program in public primary schools, year 2014 in the city of Malmö, Sweden, improved the swimming ability among children with poor socioeconomic status. Prior to this intervention, the swimming ability among children in Malmö was known to differ markedly between areas with different socioeconomic status, varying from 27% in Rosengård – a socioeconomically disadvantaged area, to 93% in Limhamn-Bunkeflo – the highest-ranked socioeconomic area in the city [5]. Pilgaard et al. found that the intervention introduced in the second grade did not decrease the socioeconomy-related differences in children’s swimming ability in the fourth grade, at least not in the first cohort exposed to the intervention based on the self-reports [4].

The process of learning to swim is presumably, like any other kind of learning, strongly affected by personal motivation [8]. Many variables, from social/environmental conditions to personal habits and internal thoughts and processes, can influence or alter a person’s motivation. According to previous literature, intrinsic motivation (often summarized as “fun” by children and youth), is the most influential factor in children’s desire to learn or participate in sports, while the extrinsic factors (i.e. achievement status, winning, pleasing parents or coaches) play generally less role as motivators [9–12]. However, intrinsic motivation does not develop independently in the child, and several external factors, such as the behaviour of the significant others (i.e. parents, relatives, good friends) can have a marked impact on children’s intrinsic motivation [10, 13]. Previous studies have reported that if children understand that sports are beneficial for health, they are more willing to participate and that children from high and medium socioeconomic backgrounds recognise more physical activity benefits than children from low socioeconomic backgrounds [14–16]. Generally, children from low socioeconomic backgrounds also experience less parental support and encouragement for physical activity than children from middle and high socioeconomic homes [15].

In the present study, we investigated to what extent certain mediating factors could explain the observed socioeconomic differences in learning how to swim among children. Among these factors were, for example, self-reported eating, sleeping and physical activity habits, as well as the characteristics of the social and working environment at both school and home. We hypothesised that variables that are generally associated with well-being, such as regular eating and sleeping habits, interest in sports and outdoor activities, active social life and general comfort at both school and home, would increase the swimming ability of children in the fourth grade.

## Methods

### Study population

This study included children who started their first-grade school-year either in August 2012 or in August 2013 at any of the public primary schools of Malmö, Sweden. Cross-sectional, self-reported questionnaire-based data about the health status and swimming ability in the fourth grade (2015/2016) were obtained from the Pupil Health Database (ELSA) with an initial sample size of 4242. We excluded those who were born outside Sweden (*n* = 708) to restrict the sample to those who have had the opportunity to learn swimming in Sweden. An additional 62 children with missing data on their swimming ability, and four children that were attending a school for autistic children were excluded. That left 3468 children as the final study cohort.

### Data collection

In Malmö, health examinations are offered to all children in preschool and in grades 4, 7 and 8 in primary schools. Since the school year of 2015/2016, school nurses enter anonymized data from health examinations at public schools (75% of all schools of Malmö) into ELSA database. For the present study, self-reported data regarding health and socioeconomic status and swimming ability (questionnaire statement “I can swim 200 meters”, answer “yes” or “no”) were retrieved from the ELSA database for the children in their fourth grade. In addition, we used a school-specific socioeconomic index (School Deprivation Index, SDI), which is regularly calculated by Statistics Sweden, and is based on following data for children at each of the schools: sex, years since arrival in Sweden (if immigrated), education of the parents, if the parents are receiving public income support, and family composition.

### Pooling and scoring of the questionnaire data

Individual-level sociodemographic variables, including participants’ family composition, parental country of origin and profession, were combined into one “Social Prerequisite Index” (SPI) (Table 1S. in Supplementary Material shows the variables and scores included into calculation of the SPI. Fig. 1S.a. in Supplementary Material displays the distribution of the variables that were pooled as SPI in relation to the probability of being able to swim). Responses to 46 questions related to health and wellbeing, retrieved from the ELSA database, were pooled into 14 potential mediator-variables by categorizing the questions according to the topics of: Eating habits, Sleeping habits, Activity level, Outdoor time, General wellbeing at school, Satisfaction with school’s physical environment, Satisfaction with school’s work environment, Satisfaction with social relations at school, Satisfaction with social relations at home, Self-satisfactions, Physical health and Negative emotions, (see Table 2S. in Supplementary Material for detailed information about the original questions included in each score). Responses to two of the statements in the questionnaire: “I am feeling well” (possible responses: “every day”, “most of the time”, “seldom”, “never”) and “My future looks bright” (possible responses: “very”, “somewhat”, “not much”, “not at all”), were estimated to weigh more than other responses and were thus evaluated as individual variables (named as “General wellbeing” and “Positivity about future”). The list of all considered mediator-variables is displayed in Table 1.

**Table 1:** Background characteristics of the study participants. Odds Ratios (OR), with confidence intervals (CI) on 95% level are shown for univariate associations between self-reported ability to swim 200 m (“yes” or “no”) and each variable

Variable name	Not able to swim 200 m (*N* = 778)	Able to swim 200 m (*N* = 2690)	or (95%CI)
Sex, n (%)			0.94 (0.80, 1.10)
Female	377 (48)	1344 (50)	
Male	401 (52)	1346 (50)	
Social Prerequisite Index (IQL), n (%)			2.28 (2.08, 2.50)
very high	71 (9)	781 (29)	
high	126 (16)	853 (32)	
low	219 (28)	622 (23)	
very low	362 (47)	434 (16)	
School-level Deprevation Index (IQL), n (%)			0.52 (0.48, 0.57)
very low	102 (13)	763 (28)	
low	100 (13)	752 (28)	
high	200 (26)	667 (25)	
very high	376 (48)	508 (19)	
*pooled health- and wellness scores devided at 50th precentile*			
Activity score, n (%)			1.31 (1.25, 1.36)
low	483 (62)	1040 (39)	
high	295 (38)	1650 (61)	
Outdoor time score, n (%)			1.52 (1.41, 1.64)
low	353 (45)	694 (26)	
high	425 (55)	1996 (74)	
Eating regularity score, n (%)			1.39 (1.30, 1.49)
low	441 (57)	1106 (41)	
high	337 (43)	1584 (59)	
Sleep score, N (%)			1.08 (1.04, 1.13)
low	456 (59)	1427 (53)	
High	322 (41)	1263 (47)	
Mental Wellbeing at school, n (%)			1.24 (1.15, 1.31)
low	410 (53)	1071 (40)	
high	368 (47)	1619 (60)	
Social relations at school, n (%)			1.17 (1.11, 1.22)
poor	421 (54)	1159 (43)	
good	357 (46)	1531 (57)	
Social relations outside school, n (%)			1.32 (1.23, 1.41)
poor	315 (40)	751 (28)	
good	463 (60)	1939 (72)	
General Wellbeing score, n (%)			1.07 (1.01, 1.13)
low	370 (48)	1159 (43)	
high	408 (52)	1531 (57)	
Self-satisfaction score, n (%)			1.07 (0.99, 1.15)
low	297 (38)	920 (34)	
high	481 (62)	1770 (66)	
Physical health, n (%)			1.00 (0.98, 1.02)
poor	382 (49)	1305 (49)	
good	396 (51)	1385 (51)	
Working environment at school, n (%)			1.14 (1.11, 1.17)
poor	500 (64)	1231 (46)	
good	278 (36)	1459 (54)	
School infrastructure satisfaction, n (%)			1.05 (1.02, 1.08)
low	364 (47)	1040 (39)	
high	414 (53)	1650 (61)	
Positivity conserning future, n (%)			1.44 (1.2, 1.64)
low	324 (42)	824 (31)	
high	454 (58)	1866 (69)	
Negative emotion score, N (%)			1.03 (0.99, 1.06)
high	370 (48)	1207 (45)	
low	408 (52)	1483 (55)	

### Statistical analysis

Swimming ability (1 = able to swim 200 m, 0 = not able to swim 200 m) was used as binary outcome variable and the interquartile levels of the SPI and SDI calculated among all included children as exposure variables. Background characteristics of the study population and crude associations between variables are shown in Table 1.

To identify potential mediator variables among the pooled health and wellbeing scores, we first explored associations between the exposure-variables (SPI and SDI) and each of the 14 pooled score variables and in linear regression analyses (Table 2S.a). Thereafter, associations between each pooled score and the outcome variable (swimming ability) and were investigated, by using logistic regression models, with adjustment for exposure variables (SPI and SDI) (Table 2S.b). Only variables associated with both the exposure and outcome at 10% statistical significance level were included in further analyses (additionally adjusted for sex). Using the more traditionally used 5% level could fail to identify important covariates, as argued by Bursac et al. [17]. In the final model (Model A, in Table 2), interactions between swimming ability and exposure- and mediator-variables were analysed by multivariable logistic regression model by using Stata 14. In Model B associations between swimming ability and exposure variables (SPI and SDI) only were analysed by multivariable logistic regression model. Post-estimation statistics were used to estimate the goodness-of-fit of the models.

**Table 2:** The estimated effect of health- and wellness-related factors mediator-variables on children’s swimming ability in association with sociodemographic variables. Odds Ratios (OR) reflect the odds of reported ability to swim 200 m (See also Fig. 1S). Model A. Association between swimming ability and sociodemographic exposures, adjusted for sex. Model B. Association between swimming ability and sociodemographic exposures, adjusted for health- and wellness-related factors and sex

Domain	Variable	Model A, OR (95%)	Model B, OR (95%)
*Sociodemographic variables*	**Sex**		
	Female	1	1
	Male	0.93 (0.78, 1.10)	0.89 (0.75, 1.06)
	**Social Prerequisite Index (IQL)**		
	Very high	5.43 (3.82, 7.73)	4.11 (2.86, 5.92)
	High	3.15 (2.41, 4.12)	2.82 (2.14, 3.72)
	Low	1.55 (1.22, 1.97)	1.13 (0.83, 1.53)
	Very low	1	1
	**School Deprivation Index (IQL)**		
	Very low	2.41 (1.80, 3.23)	2.35 (1.74, 3.18)
	Low	2.66 (2.00, 3.53)	2.67 (1.99, 3.57)
	High	1.65 (1.33, 207)	1.65 (1.32, 2.08)
	Very high	1	1
*Health- and behaviour-related mediators*	Activity score		1.19 (1.13, 1.24)
	Outdoor time score		1.19 (1.08, 1.30)
	Eating regularity score		1.05 (0.93, 1.04)
	Sleep score		0.98 (0.93, 1.04)
	Mental wellbeing at school		0.97 (0.88, 1.07)
	Work environment at school		1.03 (0.99, 1.08)
	Social relations at school		0.93 (0.86, 1.01)
	Social relations at home and during free time		1.10 (1.00, 1.20)
	Positivity about future		1.22 (1.05, 1.42)

## Results

Background characteristics of the study population are depicted in Table 1. Similar proportion of girls (78%) and boys (77%) reported that they were able to swim 200 m. Individual- and school-based sociodemographic variables (SPI and SDI) were strongly associated with children’s’ self-reported ability to swim in both Model A and Model B (Table 2, see also Fig. 1S for a graphic depiction in the Supplementary material). Nine health- and wellbeing-related variables were identified as potential mediator-variables and included in the Model B (Tables 1 and 2). Of these variables the scores for “Activity”, “Outdoor time”, “Social relationships at home and on the free time”, and “Positivity about future” were significantly and positively associated with the ability to swim (Table 2). Variables related to school environment (“Mental wellbeing at school”, “Work environment at school”, “Social relations at school”) and the individual eating and sleeping habits (“Eating regularity score”, “Sleep score”) were not associated with swimming ability in Model B. Post-estimation analyses found the sensitivity of the Model B (only including sociodemographic exposure variables) to be 94.68%, the specificity 18.77%, and the percentage of correct classification 77.65%. In comparison the sensitivity of Model A (including both sociodemographic and health- and wellbeing- related variables) was 94.50%, the specificity 24.04% and the rate of correctly classified cases 78.69%. Likelihood-ratio test after-estimation of Model A and Model B indicated a significant difference between the models (LR chi2 = 135.71; *P* < 0.001), thus indicating a better goodness of fit by model B.

## Discussion

Several factors that potentially mediate swimming ability in children of Malmö were investigated in the present study. The strong influence of socio-demographic characteristics, on both individual and school level, was evident. Among the behavioural- and lifestyle factors, scores for “Activity”, “Outdoor time”, “Social relationships at home and on the free time”, and “Positivity about future” were found to affect the relationship between socioeconomic variables and children’s swimming ability. Including both sociodemographic and health- and wellbeing-related variables, appeared to increase the fit and the percentage of correctly classified estimates in our statistical model, compared to when only sociodemographic variables were included to the model. However, the difference between models was marginal, and striking associations between individual- and school-level sociodemographic condition and swimming ability remained even after the health- and wellbeing-related factors were taken into account.

Behavioural theories, such as the Social Cognitive Theory and the Ecological Model, emphasize that health behaviours, including various physical activity skills, are not only determined by physiological and genetic characteristics of an individual, but also by socio-environmental factors, such as knowledge, self-efficacy, motivation, interpersonal relationships and possible barriers (i.e. being able to get to the location of physical activity) [18–20]. Swimming ability has been previously reported to be strongly influenced by whether the children have a parent or friends that can swim if they have knowledge of water-safety and are encouraged to swim, and if they have swimming facilities nearby [20].

Our score of “Social relationships at home and on the free time”, consisting of responses to three statements (“I like my home”, “I have friends outside the school”, and “I have an adult to talk to about important things”) was a possible indicator of children’s perception of their home environment and support network. Feeling secure and having support from one’s family, has been previously reported to be fundamental to any kind of learning processes, as well as a promotor for physical activity [21–25]. Children who felt supported by their families and friends in the present study were thus both expected and found to have higher odds of being able to swim than children without a supportive background.

The score of “Activity” was based on the responses to four questionnaire statements: “I am actively participating in physical activity (PA) lessons at school”, “I bike or walk to school”, “I am doing sports and moving a lot in my spare time”, and “I have free time hobbies (e.g. scouts, music, fishing, reading, etc.)”. Since positive responses to all these statements reflect high motivation for physical activity, experience in learning new skills and a generally active lifestyle, it was not surprising to find a significant positive relationship between this score and children’s ability to swim. Similarly, positive responses to the statements included in the “outdoor time score” (“I’m outdoors during the breaks” and “I am often outdoors after school”), are likely to reflect an individual’s habit of regular physical activity and an active lifestyle [26]. Self-efficacy – defined as “an individual’s belief in his/her capabilities to successfully execute necessary courses of action to satisfy situational demands” – has in previous literature been repeatedly identified as one of the most important factors to influence one’s will to exert and success in sports, and also, as a personal characteristic that may grow with regular physical activity [27–30]. Scoring high in “Activity” and “Outdoor time” may thus reflect and be associated to increased confidence in children about their motor capacity, which in its turn, is likely to increase an individual’s success in acquiring additional motor tasks, such as swimming ability [31].

Participants who selected “future is looking bright” (“Positivity about future”) choice had higher odds of being able to swim 200 m than those who did not agree with the statement. This finding was not surprising since expectations regarding personal abilities and future outcomes are central to the formation of human behaviour and strongly affect motivation to learn new skills [8, 31, 32]. A positive view of one’s future requires a positive appraisal of one’s current situation or a strong belief that things are going to become better. For being able to maintain such beliefs, however, a person needs both a sense of personal capability and a positive evaluation of the social environment providing the necessary support [33]. Regular participation in physical activity has been reported to reinforce the feeling of internal capability in children and leads to an increase in optimism towards a successful life [34].

Swimming proficiency may affect several aspects of the human quality of life. Besides being a good all-round way to exercise, swimming is known to alleviate symptoms in children diagnosed with various chronic conditions [35–37]. Outdoor swimming (at both natural bodies of water and at public outdoor pools) is often a social activity undertaken by groups of friends or families. Being able to swim may encourage partaking in such activities and decrease the risk of feeling excluded and socially isolated. Furthermore, sufficient swimming skills may allow partaking in more advanced water-related activities, such as snorkelling and scuba diving, which have a potential to create amazing experiences for the person. In addition, good swimming skills increase the security regarding all kinds of activities performed in the natural environments of Sweden, since water is a rather common element in the Swedish nature [38].

One way to increase children’s interest in acquiring swimming skills could be by demonstrating the wider possibilities for activates that build on good swimming skills, also in other school subjects than the physical education (i.e. in the natural and social sciences). Since the intrinsic motivation (or “fun”), is the most influential factor in children’s desire to learn physical activity skills [9–12], being able to do “fun” activities in the future may increase the motivation to work harder on their swimming skills during the lessons. However, children from families with weak socioeconomic status may not see these “fun” activities as something that is realistically available to them in the near future. Thus, besides introducing the knowledge about activities that build on good swimming skills, it may be necessary to help these children (and their families) to find a way of becoming involved in such activities despite their limited resources. The present study demonstrated that social support, optimism for the future and an active lifestyle are important for children acquiring swimming skills. Schools, municipalities and youth organisations could help develop these factors by providing opportunities for “fun” nature-based activities, involving adult instructors that can give the support and encouragement that children may need.

Previous literature, as well as the present study, has highlighted that in Sweden swimming ability is generally lower among children from families with low socioeconomic status than among children from socioeconomically stronger families [4, 6, 38]. Furthermore, in several Swedish cities a location-based pattern has been observed, according to which a lower proportion of children are able to swim in municipalities with low socioeconomic status than in municipalities with high socioeconomic status [4, 6, 38]. Thus, efforts to increase swimming proficiency in Sweden should primarily aim to increase public equality and start with acquiring location-specific knowledge that may help design such equality-increasing initiatives. According to Heckman and Masterov 2007, [39] early interventions, which partially redress the effects of adverse milieus may reverse some of the harm of disadvantage and lead to a high economic return. Thus, investing resources in activities that increase children’s intrinsic motivation for an active life in socioeconomically weak areas may, not only benefit the children themselves, but also society at large by decrease future costs of, for example, legal system and health care.

The strengths of the present study were the availability of data on sociodemographic factors both at the individual and the school level and the relatively large sample included. In addition, the participation in the health examinations entered in the Pupil Health Database was high, which limited the risk of selection bias. However, using self-reported data and a cross-sectional study design were significant limitations of our study. Since no documented school grades concerning swimming ability were available, it is possible that some children may have over- or underestimated their swimming skills. However, it is reasonable to assume that the misclassification regarding over- and underestimation of one’s swimming ability was non-differential. There is also a potential for reverse causality since individuals who can swim, maybe generally more active which may result in increased wellbeing and positivity. A prospective cohort study, also including data on attitudes towards swimming as well as engagement and swimming ability among the parents, would elucidate drivers as well as barriers to swimming ability among children further.

## Conclusions

Socioeconomic factors are strongly associated with children’s swimming ability. Lower socioeconomic status at both individual and school level is associated with lower odds of swimming anility among children. While most of the sociodemographic gradient in swimming ability remained unexplained in our study, social support, optimism for the future and an active lifestyle, however, were associated with sociodemographic conditions and also positively associated with children’s swimming skills. Interventions should thus, in addition to children’s swimming lessons, target the whole families with the goal of increasing their possibilities for socialising and engaging in different kinds of recreational activities especially in lower socioeconomic neighbourhoods. Swimming, as an activity has many positive health effects, both physiological and mental. Learning of these benefits may influence children’s will to acquire swimming skills. Having “fun” (defined as a positive mood state related to personal achievement and perceptions that their skills are matched against realistic challenges) is, however, the most commonly reported intrinsic motivator by children and youth for participating in different kinds of sports and should therefore be in focus when designing the interventions. Our findings can be used to target groups of children both at individual- and school-level with greater needs for support in swimming training.

## Supplementary Information

**Additional file 1.**

## Data Availability

The data that support the findings of this study are available from Pupil Health Database (ELSA), but restrictions apply to the availability of these data, which were used under license for the current study, and so are not publicly available. Data are however available from the authors upon reasonable request and with permission of the National Ethics Review Board.

## Abbreviations

CI: Confidence Interval
ELSA: the Pupil Health Database (*Elevhälsodatabasen*)
IQL: Inter-Quartile Level
OR: Odds Ratio
PA: Physical Activity
SDI: School Deprivation Index
SP: Social Prerequisite Index
