# Supplementary material for: What makes children learn how to swim? – health, lifestyle and environmental factors associated with swimming ability among children in the city of Malmö, Sweden
Source: BMC Pediatr. 2022 Jan 10;22:32. doi: 10.1186/s12887-021-03094-0 (PMC8744283; doi:10.1186/s12887-021-03094-0)
Supplement: Supplementary file 1 — Additional file 1. [file 12887_2021_3094_MOESM1_ESM.docx]

## Supplementary material

Table 1s. Factors included in the Social Prerequisite Index (SPI).

| Question | RESPONSE Alternatives | | | | |
| --- | --- | --- | --- | --- | --- |
| The child lives with… | Both parents |  | Alternates between parents |  | Single parent or not with parents |
| scoring value | 1 |  | 3 |  | 5 |
| The occupation of the parents | Both non-manual labour | One non-manual one other | Both manual labour | One manual one not working | Both presently not working |
| scoring value | 1 | 2 | 3 | 4 | 5 |
| The country of birth of the parents | Both Swedish/ Nordic | One Swedish/ Nordic one other | One or both from rest of the Europe, North America or Australia | Both outside Europe, North America or Australia |  |
| scoring value | 1 | 2 | 3 | 4 |  |
| IQL of the SPI scores (min=3; max=14) | Very high | High | Low | Very low |  |
| Range | 3 | 4-6 | 7-8 | 9-14 |  |

Table 2s. Description of the scoring method. The name of each new health- and wellness-related variable (1) and the questions included (column 2)

|  | Question | How right is it in my case? | | | |
| --- | --- | --- | --- | --- | --- |
|  |  | **Very** | **Some-what** | **Not much** | **Not at all** |
| Eating Score | I eat breakfast every morning | 4 | 3 | 2 | 1 |
|  | I eat school lunch every school day | 4 | 3 | 2 | 1 |
|  | I eat dinner/supper every day | 4 | 3 | 2 | 1 |
| (min, max) |  | 12 |  |  | 3 |
| Sleeping Score | It is easy for me to fall asleep in the evening | 4 | 3 | 2 | 1 |
|  | I sleep well at night | 4 | 3 | 2 | 1 |
|  | I feel rested when I wake up in the morning | 4 | 3 | 2 | 1 |
| (min, max) |  | 12 |  |  | 3 |
| activity score | I am actively participating in PA lessons at school | 4 | 3 | 2 | 1 |
|  | I bike or walk to school | 4 | 3 | 2 | 1 |
|  | I am doing sports and moving a lot in my spare time | 4 | 3 | 2 | 1 |
|  | I have free time hobbies (e.g. scouts, music, fishing, reading, etc.) | 4 | 3 | 2 | 1 |
| (min, max) |  | 16 |  |  | 4 |
| Outdoor time score | I'm outdoors during the breaks | 4 | 3 | 2 | 1 |
|  | I am often outdoors after school | 4 | 3 | 2 | 1 |
| (min, max) |  | 8 |  |  | 2 |
| Wellbeing at school | I feel calm and confident about going to school | 4 | 3 | 2 | 1 |
|  | I like being at school | 4 | 3 | 2 | 1 |
| (min, max) |  | 8 |  |  | 2 |
| School infrastructure satisfaction | I like my schoolyard | 4 | 3 | 2 | 1 |
|  | I think my school's toilets are clean | 4 | 3 | 2 | 1 |
|  | I think the changing rooms and showers at school are functioning and clean | 4 | 3 | 2 | 1 |
|  | I think the environment in the school canteen is good | 4 | 3 | 2 | 1 |
| (min, max) |  | 16 |  |  | 4 |
| School work environment score | I can work in peace during the lessons | 4 | 3 | 2 | 1 |
|  | I think the teachers listen to me | 4 | 3 | 2 | 1 |
|  | I get the help I need at school | 4 | 3 | 2 | 1 |
|  | I have no difficulties with school work | 4 | 3 | 2 | 1 |
|  | I am happy with my school work | 4 | 3 | 2 | 1 |
|  | I feel involved when deaccessions are made at the school | 4 | 3 | 2 | 1 |
| (min, max) |  | 24 |  |  | 6 |
| School relations Score | I have friends in school | 4 | 3 | 2 | 1 |
|  | I think all the staff in the school are kind to me | 4 | 3 | 2 | 1 |
|  | I think all students at school are kind to me | 4 | 3 | 2 | 1 |
|  | I am friendly to all students and staff at school | 4 | 3 | 2 | 1 |
| (min, max) |  | 16 |  |  | 4 |
| home and free time relations score | I like my home | 4 | 3 | 2 | 1 |
|  | I have friends outside the school | 4 | 3 | 2 | 1 |
|  | I have an adult who I can talk to about important things | 4 | 3 | 2 | 1 |
| (min, max) |  | 12 |  |  | 3 |
| Positivity About Future | My future looks bright | 4 | 3 | 2 | 1 |
| (min, max) |  | 4 |  |  | 1 |

|  | Question | How often does it happen? | | | |
| --- | --- | --- | --- | --- | --- |
|  |  | **Every day** | **Most of the time** | **Seldom** | **Never** |
| General Wellbeing | I am feeling well | 4 | 3 | 2 | 1 |
| (min, max) |  | 4 |  |  | 1 |
| Self-satisfaction score | I feel satisfied with myself | 4 | 3 | 2 | 1 |
|  | I'm happy with my body | 4 | 3 | 2 | 1 |
| (min, max) |  | 8 |  |  | 2 |
| Negative emotion score | I feel sad | 1 | 2 | 3 | 4 |
|  | I feel worried | 1 | 2 | 3 | 4 |
|  | I feel tired during daytime | 1 | 2 | 3 | 4 |
|  | I feel annoyed or in a bad mood | 1 | 2 | 3 | 4 |
|  | I feel angry | 1 | 2 | 3 | 4 |
| (min, max) |  | 5 |  |  | 20 |
| Physical health score | I have a headache | 1 | 2 | 3 | 4 |
|  | I feel dizzy | 1 | 2 | 3 | 4 |
|  | I have stomach ache | 1 | 2 | 3 | 4 |
|  | I have back, neck or shoulder pain | 1 | 2 | 3 | 4 |
|  | I have pain in my hips, knees or feet | 1 | 2 | 3 | 4 |
|  | I need medicine/drugs | 1 | 2 | 3 | 4 |
| (min, max) |  | 6 |  |  | 24 |

**Fig 1S.** Groups based on the interquartile level (IQL) of the estimated probability swimming ability (1^st^ quartile – “Very low”, 2^nd^ quartile – “Low”, 3^rd^ quartile – “High” and 4^th^ quartile – “Very high”).

Swimming probability groups were calculated based on models A. (left) and B. (right, see also Table 2). **a)** Distribution (%) of the variables included in “Social Prerequisite Index” (SPI) in the swimming probability groups. **b)** Distribution (%) of IQL school-lever deprivation index and IQL SPI within different probability groups

a)


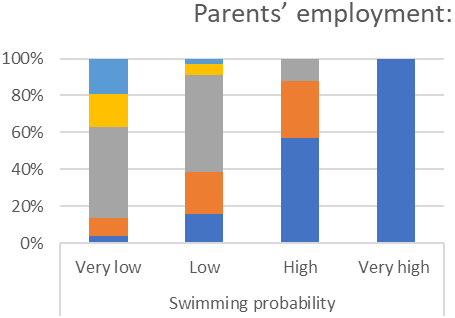

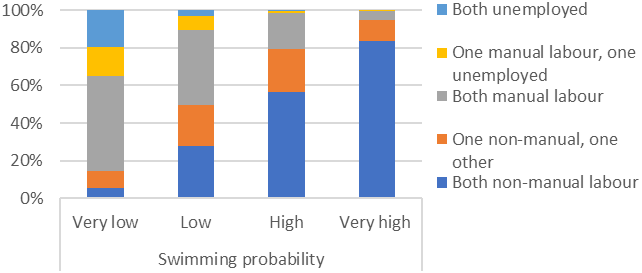

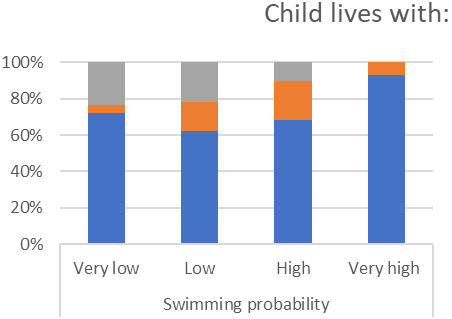

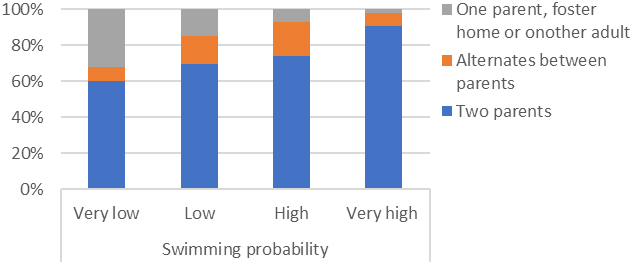

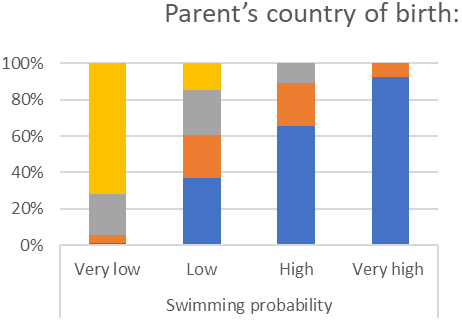

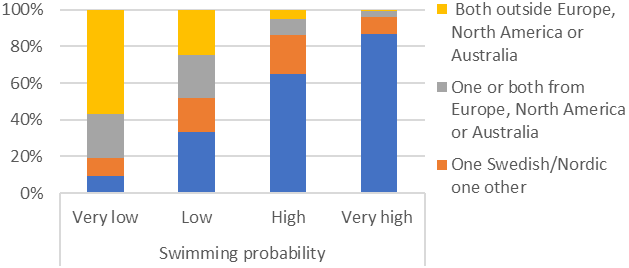


b)


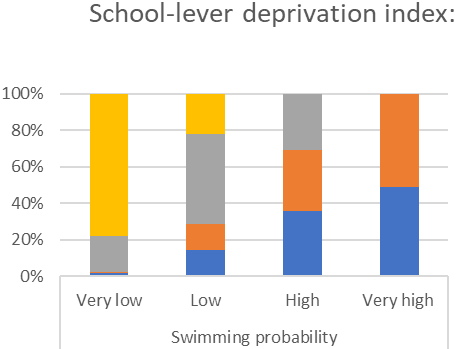

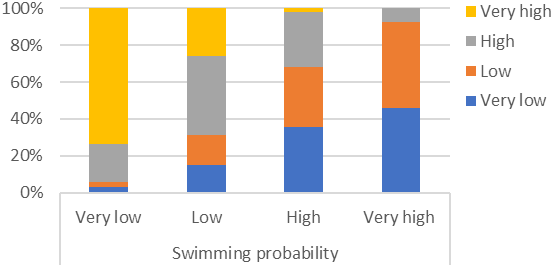

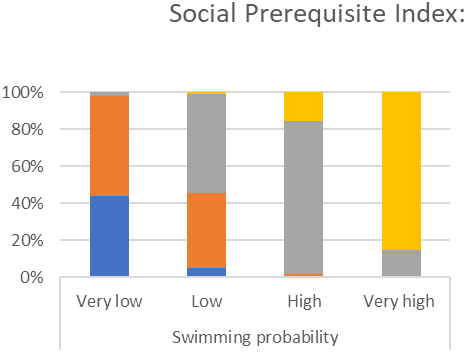

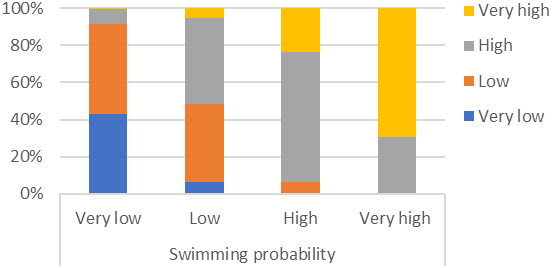


Table 2S. Exploratory statistics

a) Association between a possible single mediator and exposure variables (IQLs of social prerequisite index, SPI, and school deprivation index, SDI).

|  | SPI, β (95% CI) | SDI, β (95% CI) |
| --- | --- | --- |
| Activity | 0.35 (0.28, 0.43) | -0.07 (-0.14, 0.03) |
| Outdoor time | 0.13 (0.09, 0.17) | -0.09 (-0.12, -0.05) |
| Eating regularity | 0.25 (0.21, 0.30) | -0.10 (-0.14, -0.06) |
| Sleep | 0.10 (0.31, 0.18) | 0.02 (-0.04, 0.08) |
| Mental wellbeing at school | 0.08 (0.03, 0.12) | -0.15 (-0.19, -0.11) |
| School Infrastructure satisfaction | 0.18 (0.08, 0.29) | -0.30 (-0.39, -0.21) |
| School relations | 0.24 (0.17, 0.31) | -0.21 (-0.27, -0.15) |
| Home and free time relations | 0.15 (0.11, 0.20) | -0.02 (-0.06, 0.02) |
| General wellbeing | 0.07 (0.01, 0.13) | 0.01 (-0.04, 0.06) |
| Negative emotions | -0.06 (-0.17, 0.05) | -0.06 (-0.16, 0.03) |
| Physical health | 0.09 (-0.07, 0.25) | 0.11 (-0.02, 0.25) |
| School work environment | 0.40 (0.28, 0.51) | -0.33 (-0.43, -0.23) |
| Positivity about future | 0.03 (0.01, 0.06) | 0.01 (-0.01, 0.03) |
| Self-satisfaction | 0.04 (0.03, 0.10) | 0.00 (-0.04, 0.04) |

b) Association between outcome and a potential single mediator, adjusted for IQL of the exposure variables

|  | Main,  OR (95% CI) | SPI,  OR (95% CI) | SDI,  OR (95% CI) |
| --- | --- | --- | --- |
| Activity | 1.24 (1.19, 1.30) |  |  |
| Very low |  | Ref | Ref |
| Low |  | 1.52 (1.19, 1.95) | 1.13 (0.84, 1.54) |
| High |  | 3.00 (2.28, 3.93) | 0.70 (0.53, 0.93) |
| Very high |  | 4.31 (3.10, 6.33) | 0.42 (0.31, 0.57) |
| Outdoor time | 1.33 (1.23, 1.44) |  |  |
| Very low |  | Ref | Ref |
| Low |  | 1.46 (1.14, 1.87) | 1.09 (0.81, 1.47) |
| High |  | 2.96 (2.26, 3.89) | 0.73 (0.55, 0.97) |
| Very high |  | 4.93 (3.45, 7.03) | 0.44 (0.33, 0.60) |
| Eating regularity | 1.17 (1.09, 1.26) |  |  |
| Very low |  | Ref | Ref |
| Low |  | 1.51 (1.18, 1.92) | 1.13 (0.84, 1.52) |
| High |  | 2.92 (2.23, 3.83) | 0.72 (0.54, 0.95) |
| Very high |  | 4.90 (3.40, 6.94) | 0.43 (0.32, 0.58) |
| Sleep | 1.08 (1.03, 1.13) |  |  |
| Very low |  | Ref | Ref |
| Low |  | 1.53 (1.20, 1.95) | 1.11 (0.83, 1.51) |
| High |  | 3.09 (2.36, 4.04) | 0.69 (0.52, 0.92) |
| Very high |  | 5.29 (3.72, 7.53) | 0.41 (0.31, 0.55) |
| Mental wellbeing at school | 1.11 (1.03, 1.19) |  |  |
| Very low |  | Ref | Ref |
| Low |  | 1.55 (1.22, 1.98) | 1.12 (0.83, 1.51) |
| High |  | 3.14 (2.40, 4.11) | 0.71 (0.54, 0.94) |
| Very high |  | 5.34 (3.75, 7.59) | 0.43 (0.32, 0.58) |
| School work environment | 1.08 (1.05, 1.11) |  |  |
| Very low |  | Ref | Ref |
| Low |  | 1.53 (1.20, 1.95) | 1.14 (0.84, 1.54) |
| High |  | 3.04 (2.32, 3.98) | 0.73 (0.55, 0.97) |
| Very high |  | 5.02 (3.52, 7.16) | 0.44 (0.33, 0.60) |
| School relations | 1.05 (0.10, 1.10) |  |  |
| Very low |  | Ref | Ref |
| Low |  | 1.54 (1.21, 1.96) | 1.11 (0.82, 1.50) |
| High |  | 3.10 (2.37, 4.06) | 0.71 (0.53, 0.93) |
| Very high |  | 5.26 (3.69, 7.49) | 0.43 (0.32, 0.57) |
| Home and free time relations | 1.22 (1.13, 1.31) |  |  |
| Very low |  | Ref | Ref |
| Low |  | 1.54 (1.21, 1.96) | 1.63 (0.83, 1.52) |
| High |  | 3.02 (2.30, 3.96) | 0.55 (0.53, 0.92) |
| Very high |  | 5.01 (3.52, 7.14) | 0.23 (0.31, 0.56) |
| Self-satisfaction | 1.04 (0.96, 1.12) |  |  |
| Very low |  | Ref | Ref |
| Low |  | 1.54 (1.21, 1.96) | 1.10 (0.82, 1.48) |
| High |  | 3.14 (2.40, 4.11) | 0.68 (0.52, 0.91) |
| Very high |  | 5.40 (3.80, 7.69) | 0.41 (0.31, 0.56) |
| Physical health | 1.01 (0.98, 1.03) |  |  |
| Very low |  | Ref | Ref |
| Low |  | 1.55 (1.22, 1.97) | 1.10 (0.82, 1.49) |
| High |  | 3.15 (2.42, 4.13) | 0.69 (0.52, 0.91) |
| Very high |  | 5.42 (3.81, 7.72) | 0.41 (0.31, 0.55) |
| School Infrastructure satisfaction | 0.99 (0.96, 1.03) |  |  |
| Very low |  | Ref | Ref |
| Low |  | 1.54 (1.22, 1.97) | 1.17 (0.82, 1.48) |
| High |  | 3.16 (2.42, 4.14) | 0.68 (0.52, 0.90) |
| Very high |  | 5.45 (3.83, 7.76) | 0.41 (0.31, 0.55) |
| Positivity about future | 1.43 (1.25, 1.64) |  |  |
| Very low |  | Ref | Ref |
| Low |  | 1.55 (1.21, 1.97) | 1.10 (0.81, 1.48) |
| High |  | 3.13 (2.39, 4.10) | 0.67 (0.51, 0.89) |
| Very high |  | 5.30 (3.72, 7.54) | 0.41 (0.30, 0.54) |
| General wellbeing | 1.05 (0.99, 1.11) |  |  |
| Very low |  | Ref | Ref |
| Low |  | 1.55 (1.21, 1.97) | 1.10 (0.82, 1.49) |
| High |  | 3.14 (2.40, 4.11) | 0.69 (0.52, 0.91) |
| Very high |  | 5.38 (3.78, 7.65) | 0.41 (0.31, 0.55) |
| Negative emotions | 1.03 (1.00, 1.06) |  |  |
| Very low |  | Ref | Ref |
| Low |  | 1.55 (1.22, 1.98) | 1.10 (0.82, 1.49) |
| High |  | 3.19 (2.44, 4.18) | 0.69 (0.52, 0.91) |
| Very high |  | 5.45 (3.84, 7.76) | 0.44 (0.31, 0.56) |
